# Treating addiction with an addictive drug: the ketamine paradox revisited

**DOI:** 10.3389/fpsyt.2026.1866092

**Published:** 2026-07-09

**Authors:** Alqassem Y. Hakami

**Affiliations:** 1 College of Medicine, King Saud Bin Abdulaziz University for Health Sciences, Jeddah, Saudi Arabia; 2 King Abdullah International Medical Research Center, Jeddah, Saudi Arabia

**Keywords:** addiction, esketamine, ketamine, substance use disorder, treatment-resistant depression (TRD)

## Abstract

**Background:**

Substance use disorders (SUDs) and treatment-resistant depression (TRD) remain a major global health challenge, marked by high relapse rates and limited long-term effectiveness of existing treatments. Ketamine, a glutamatergic modulator with rapid neuroplastic effects, has emerged as a novel intervention for TRD and is increasingly investigated as an adjunctive treatment for addiction, yet concerns about its abuse liability persist.

**Objective:**

This review critically evaluates ketamine’s therapeutic potential for SUDs while examining its neurobiological mechanisms, clinical efficacy, and risk of misuse within a unified risk–benefit framework.

**Methods:**

A structured narrative review conducted in accordance with the SANRA framework using the PubMed, Scopus, PsycINFO, and Web of Science databases, covering literature published until March 2026. Eligible studies included clinical trials, experimental studies, and mechanistic investigations relevant to ketamine use in addiction and depression. Evidence was synthesized thematically across the domains of efficacy, mechanisms, and safety.

**Results:**

Across alcohol and cocaine use disorders, ketamine combined with psychotherapy has demonstrated promising reductions in craving and increases in abstinent days in small-to moderate-sized Phase 2 trials. However, findings remain difficult to generalize due to considerable variability in dosing strategies, comparator conditions, and follow-up periods. Effects on relapse prevention have been more inconsistent and less reliably positive. Mechanistically, ketamine promotes synaptic plasticity via NMDA receptor antagonism and downstream glutamatergic signaling, potentially disrupting maladaptive reward-related memories and reversing maladaptive neurocircuitry involved in both depression and addiction. While acute adverse effects are generally transient under clinical supervision, ketamine carries a well-established risk of misuse, particularly in unsupervised or high-dose settings.

**Conclusions:**

Ketamine represents a promising but still experimental intervention for both refractory depression and selected SUDs. Its clinical use depends on careful patient selection, structured delivery, and integration with psychotherapy. Although ketamine may redefine treatment paradigms for TRD and addiction, larger-scale trials and long-term safety data are essential to define its role within psychiatric and addiction treatment frameworks.

## 1 Introduction

### The global burden of addiction

Addiction is a chronic, relapsing disorder characterized by compulsive drug seeking and use despite adverse consequences, supported by durable neuroadaptations in mesocorticolimbic reward, anti-reward and stress, and prefrontal executive control circuits (1, 2). These neurobiological changes involve dysregulation of mesocorticolimbic dopamine, impairments in inhibitory control in the prefrontal cortex, and synaptic plasticity in the nucleus accumbens, amygdala, and prefrontal cortex, thus establishing a long-lasting vulnerability to relapse (3–5). Epidemiologically, substance use disorders contribute substantially to global morbidity and mortality and have shown rising harms in many regions, including increases in overdose deaths and treatment demand (1, 6). The burden extends beyond health impacts to include major economic costs from lost productivity and some criminal impacts (1, 5, 7).

### Treatment limitations and relapse

Despite advances in addiction treatment, current pharmacological and behavioral interventions still leave significant gaps in effectiveness and long-term benefits. Current medications such as methadone, naltrexone, and buprenorphine are used to reduce opioid use and overdose harm, but they may face access barriers as well as limited effectiveness for comorbidities and multisubstance use (1, 8). Behavioral treatments like cognitive-behavioral therapy and contingency management can improve coping skills and abstinence. However, effectiveness varies, implementation is inconsistent, and maintaining long-term gains is challenging (4, 9, 10). Relapse remains one of the main barriers to the successful interventions of substance use disorders (11, 12). Treatment gaps involve a lack of personalized targeting for neurobiological issues and poor integration of medication and behavioral therapies. These unmet needs drive the development of new interventions based on mechanisms and biomarker-guided strategies to enhance retention and prevent relapse (1, 7, 13).

In addition, among alcohol use disorders (AUD), three medications are widely approved by the Food and Drug Administration/European Medicines Agency (FDA/EMA), including disulfiram, naltrexone, and acamprosate. Yet they are modestly effective and less widely used (14–19). Current guidelines recommend oral naltrexone and acamprosate as first−line pharmacotherapies, with disulfiram used more selectively, typically alongside psychosocial care (14, 15, 19). In addition, for tobacco use disorder, several pharmacotherapies are established. Varenicline, nicotine replacement therapy (NRT), and bupropion are licensed first−line smoking−cessation aids, with varenicline and combination NRT showing the highest quit rates, especially when combined with behavioral counseling (20–23). By contrast, for psychostimulant and cannabis use disorders, there are still no FDA/EMA−approved pharmacotherapies. For methamphetamine/amphetamine use disorder, multiple agents (including stimulants, antidepressants, anticonvulsants, naltrexone, and varenicline) have been tested, but no medication has shown sufficient, consistent benefit to achieve regulatory approval (24–29). Similarly, in cocaine use disorder, no pharmacotherapy is approved in the US or Europe, and large meta−analyses highlight the absence of any effective standard medication, in contrast to approved treatments for opioid, alcohol, and tobacco use disorders (25, 30). Collectively, these reviews of current status and future directions underscore a major unmet need for effective, approved medications for cocaine, methamphetamine, and cannabis addictions, in striking contrast to the pharmacotherapy armamentarium available for opioid, alcohol, and tobacco use disorders (25, 28–30).

### Ketamine as a potential treatment: a unique mechanism

Interest in repurposing the use of ketamine for addiction therapy has increased over the last decade, driven by limited success with traditional methods (31, 32). Ketamine is characterized by mechanisms that promote increased neuroplasticity and synaptic remodeling. These effects have been documented previously at cellular and molecular levels in reviews of psychedelics and similar compounds (33). It has been proposed that ketamine modulates the glutamatergic system by antagonizing N-methyl-D-aspartate (NMDA) receptors (34, 35). Unlike classical psychedelics such as psilocybin and Lysergic acid diethylamide (LSD), which act primarily as 5-HT₂A receptor agonists, ketamine is mechanistically distinct, functioning mainly as an uncompetitive NMDA receptor antagonist with additional activity at α-amino-3-hydroxy-5-methyl-4-isoxazolepropionic acid (AMPA), opioid (μ and κ), and muscarinic receptors (36). The active metabolite (2R,6R)-hydroxynorketamine (HNK) exerts antidepressant effects through AMPA receptor potentiation via an NMDA-independent pathway, as demonstrated by Zanos et al. (37), with implications for understanding the dissociability of therapeutic from psychotomimetic effects. Enantiomeric divergence is also clinically relevant: (S)-ketamine (esketamine) has higher NMDA receptor affinity and forms the basis of the only FDA-approved intranasal formulation, whereas (R)-ketamine shows antidepressant efficacy in preclinical models with potentially fewer dissociative effects (38). Glutamatergic dysfunction is not confined to addiction neurocircuitry: converging evidence implicates NMDA receptor hypofunction and disrupted excitatory–inhibitory balance in the pathophysiology of major depressive disorder and, critically, in suicidality. Esketamine’s capacity to rapidly normalize synaptic glutamate signaling in prefrontal and limbic circuits underpins both its FDA approval for treatment-resistant depression and its emerging relevance for suicidal ideation in acute psychiatric presentations (39). This shared glutamatergic substrate linking addiction, depression, and suicidality strengthens the mechanistic rationale for ketamine’s cross-diagnostic utility and contextualizes the comorbidity framing developed in Section 3.5. Overall, reviews indicate that these substances provide multiple biological options to significantly reshape reward and learning pathways involved in relapse and compulsive behavior, encouraging more rigorous trials and integrated psychotherapeutic approaches (40).

### Current problem and research aim

Despite growing interest in ketamine as a potential treatment for substance use disorders, the current literature remains divided between mechanistic studies, small clinical trials, and disorder-specific investigations. While several systematic reviews have demonstrated efficacy and safety considerations in the ketamine literature (41–43), a unified narrative synthesis that simultaneously evaluates neurobiological mechanisms, clinical evidence across multiple SUD subtypes, and abuse liability within a structured risk–benefit framework remains absent from the SUD-specific literature.

Critically, there is a lack of comprehensive frameworks that simultaneously evaluate ketamine’s neurobiological mechanisms, clinical effectiveness, and abuse liability within the same translational context. In particular, insufficient attention has been given to distinguishing therapeutic use under controlled conditions from patterns of misuse, as well as to identifying patient-level factors that may shift the balance between benefit and risk. Although ketamine shows promising therapeutic potential for substance abuse treatment, the risk of abuse remains a significant side effect that could complicate its clinical use (44, 45). As a result, clinicians and researchers currently lack clear guidance on how to position ketamine within existing treatment paradigms for addiction.

This review aims to address these gaps by integrating evidence across neurobiological, clinical, and safety domains to provide a structured risk–benefit evaluation of ketamine in the treatment of substance use disorders. The aim of this review is to critically assess the therapeutic potential of ketamine in addiction treatment, while addressing risks of misuse and safety concerns. This aims to guide research priorities, clinical practices, and public health strategies.

## 2 Methods

This review offers a comprehensive overview of the current literature on Ketamine in treating substance use disorders, using a selective approach to identify and assess the most relevant evidence in this rapidly evolving field.

### 2.1 Literature search strategy

This review uses a narrative approach to synthesize the rapidly evolving and methodologically heterogeneous literature on ketamine for substance use disorders. A formal systematic review or meta-analysis was not conducted because the available evidence spans diverse study designs, including small randomized controlled trials, pilot studies, laboratory-based experiments, observational reports, and mechanistic preclinical research, often with substantial variability in patient populations, dosing regimens, psychotherapeutic integration, and outcome measures. This heterogeneity limits the feasibility of quantitative pooling and reduces the interpretability of aggregate effect estimates.

Moreover, the aims of this structured narrative review extend beyond evaluating clinical efficacy to integrating neurobiological mechanisms, psychotherapeutic frameworks, and safety considerations, particularly abuse liability and long-term risks. Such multidimensional synthesis is better suited to a narrative methodology, which allows for contextual interpretation of findings across translational domains rather than strict aggregation of outcomes.

To enhance transparency and rigor, a structured literature search strategy, predefined inclusion and exclusion criteria, and thematic organization of evidence were employed. This approach enables a comprehensive and clinically meaningful synthesis while acknowledging the limitations inherent to non-systematic methodologies. Importantly, this approach is consistent with established guidance for narrative reviews addressing complex, emerging therapeutic areas where evidence is insufficiently standardized for formal meta-analytic techniques.

**Figure 1:**
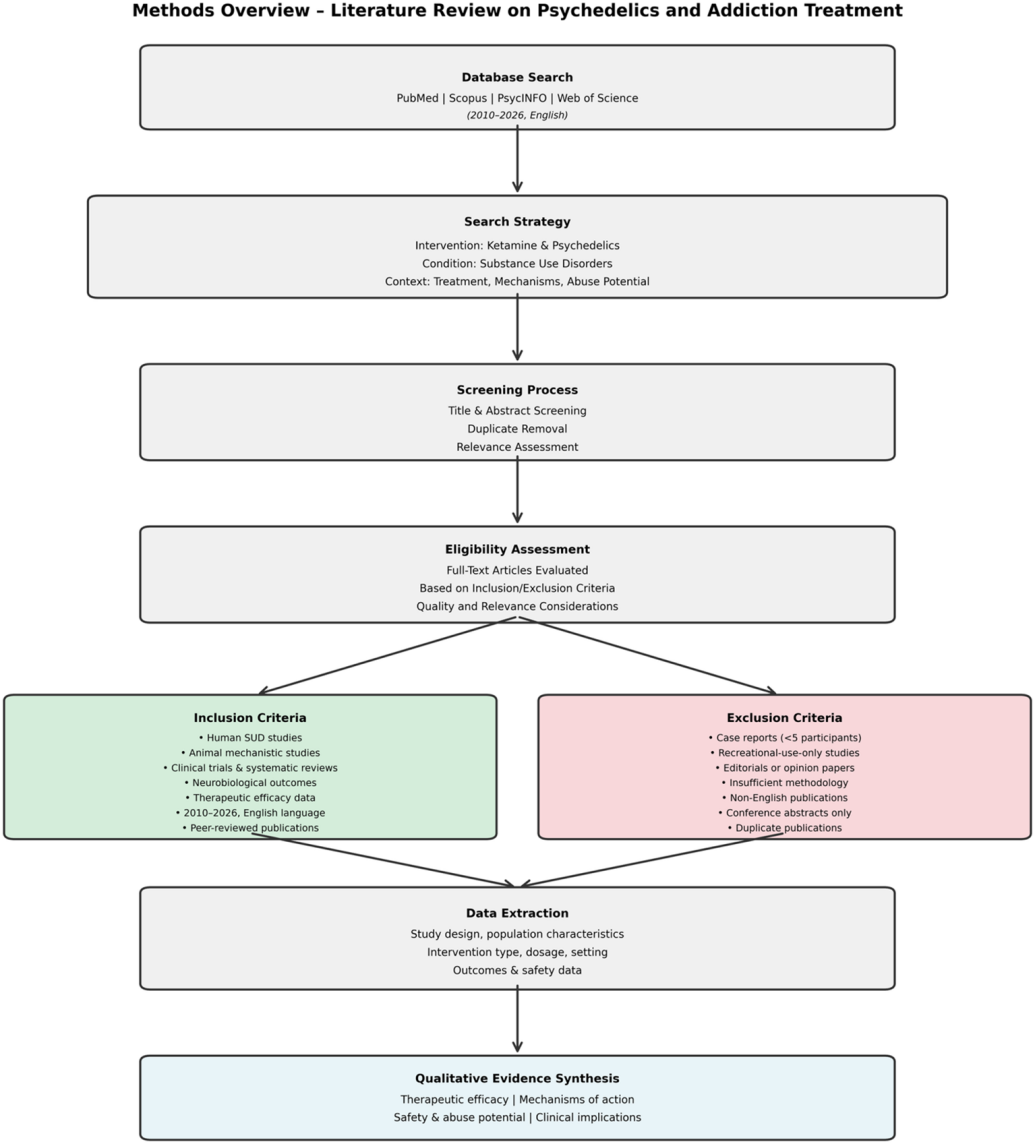
Illustrates the methodological framework diagram of the structured narrative review process (more details on the exclusion criteria available in section 2.1).

The methodological framework diagram for this structured narrative review (outlined in **Figure 1**) was conducted according to (46) and checked against six Scale for the Assessment of Narrative Review Articles (SANRA) quality items: (1) relevance and rationale for the narrative review, (2) literature search strategy, (3) statement of review aims, (4) appropriate referencing, (5) scientific reasoning, and (6) presentation of data. A completed SANRA checklist has been appended as **Supplementary Table 1**. A comprehensive literature search was conducted across four major electronic databases: PubMed (MEDLINE), Scopus, PsycINFO, and Web of Science Core Collection.

The review process went through several stages of screening and evaluation. Initial screening of titles and abstracts with duplicate removal was followed by a full-text eligibility assessment based on predefined inclusion and exclusion criteria consistent with established practices in narrative review methodology (47, 48). Inclusion criteria focused on human clinical studies, animal mechanistic research, systematic reviews, and neurobiological investigations published until 15 March 2026, while exclusion criteria ruled out case reports, recreational-use-only studies, and publications lacking sufficient methodological rigor. Case reports with fewer than 5 participants were excluded from the efficacy synthesis. For the safety synthesis, case reports and small series were retained where they documented adverse events that are underrepresented in clinical trial populations, specifically hepatobiliary and uropathic toxicity. This split approach is consistent with previous review literature on ketamine safety (49, 50). Moreover, excluded from the efficacy synthesis: studies reporting only recreational ketamine use without any therapeutic administration context. In addition, retained for the harms synthesis: studies of recreational use patterns relevant to characterising abuse liability, dose-escalation profiles, dependence prevalence, and adverse event profiles in consistency with the approach used in systematic and scoping reviews of ketamine abuse liability (43, 51).

Data extraction from eligible studies involved capturing key characteristics, including study details, population demographics, intervention parameters, and outcome measures. The final stage consisted of qualitative evidence synthesis, where findings were organized thematically to examine therapeutic efficacy, underlying mechanisms, safety considerations, and potential for abuse. This structured approach provided thorough coverage of the literature, focusing on studies most relevant to understanding how ketamine is used therapeutically in addiction treatment. The list of search terms is available in **Table 1**.

#### 2.1.1 Search terms and database queries

Search strategies were adapted to the indexing conventions of each database (PubMed/MEDLINE, Scopus, PsycINFO, Web of Science). Searches were built using the intervention, condition, and context terms listed in **Table 1**, combined as appropriate for each database's search interface. Searches were limited to peer-reviewed, English-language publications from 2010 to 15 March 2026. Reference lists of key reviews were hand-searched to identify additional eligible studies.

**Table 1:** Lists the search terms used for data collection across three domains: intervention terms, condition terms, and context terms.

Intervention terms	Condition terms	Context terms
“ketamine”	“addiction”	“treatment”
“(S)-ketamine (esketamine)”	“substance use disorder”	“therapy”
“(R)-ketamine (arketamine)”	“substance abuse”	“intervention”
“hydroxynorketamine (HNK)”	“substance dependence”	“clinical trial”
“(2R,6R)-HNK”“hydroxynorketamine (HNK)”	“alcohol use disorder”	“efficacy”
“ketamine-assisted psychotherapy (KAP)”	“alcohol dependence”	“effectiveness”
“ketamine psychotherapy (KPT)”	“opioid use disorder”	“therapeutic”
	“opioid dependence”	“abuse potential”
	“heroin addiction”	“mechanism*”
	“cocaine dependence”	“neurobiology”
	“stimulant use disorder”	“pharmacology”
	“cannabis use disorder”	“NMDA antagonist”
	“drug abuse”	“glutamatergic”
		“AMPA”
		“BDNF”
		“TrkB”
		“mTOR”
		“neuroplasticity”
		“memory reconsolidation”
		“synaptic remodelling”
		“self-administration”
		“uropathy”
		“cystitis”
		“bladder”
		“hepatotoxicity”
		“ketamine use disorder”
		“dependence”
		“abuse liability”
		“dissociation”
		“cholangiopathy”

## 3 Neurobiological rationale

### 3.1 Ketamine: NMDA receptor antagonism, glutamatergic modulation, and synaptic plasticity

Ketamine is a non-competitive, open-channel antagonist at ionotropic NMDA receptors, which are essential for synaptic plasticity, learning, and memory. Unlike classical serotonergic psychedelics such as psilocybin and LSD, which act primarily as 5-HT₂A receptor agonists, ketamine is mechanistically unique: its primary pharmacological target is the glutamatergic system, with secondary activity at AMPA receptors, opioid (μ and κ) receptors, HCN1 channels, and muscarinic receptors (36).

At sub-anesthetic doses, partial NMDA blockade, acting preferentially on GABAergic interneurons, disinhibits pyramidal cells and transiently increases glutamate release. This activates AMPA receptors and downstream BDNF-TrkB-mTOR signaling pathways, leading to rapid synaptogenesis and enhanced excitatory transmission. In the context of addiction, the relevance of this mechanism extends beyond antidepressant action: maladaptive synaptic strengthening in the nucleus accumbens, prefrontal cortex, and amygdala consolidates drug-related cue–reward associations that drive craving and relapse. Ketamine-induced synaptogenesis in these circuits may reverse pathological engrams and restore prefrontal inhibitory control over drug-seeking behavior (52, 53).

### 3.2 Hydroxynorketamine and AMPA-mediated, NMDA-independent effects

The active metabolite (2R,6R)-HNK has emerged as a pharmacologically important entity as demonstrated in Zanos et al. study, which shows that (2R,6R)-HNK exerts antidepressant effects through AMPA receptor potentiation via an NMDA-independent pathway, challenging the assumption that NMDA receptor blockade is necessary for ketamine’s therapeutic actions (37). Recent work confirmed that blocking Ca²⁺-permeable AMPA receptors abolishes ketamine’s rapid antidepressant-like effects in animal models (54).

For addiction treatment, the (2R,6R)-HNK pathway is clinically significant because it may contribute to therapeutic benefit independently of the dissociative and psychotomimetic effects mediated by NMDA blockade, raising the possibility that this metabolite or its analogs could separate therapeutic from abuse-liability-generating mechanisms. Second, it provides a rationale for the sustained effects of ketamine beyond its short plasma half-life, since HNK accumulates and remains in the system longer than the parent compound (55).

### 3.3 Enantiomer divergence: (S)-ketamine and (R)-ketamine

Racemic ketamine is administered as an equal mixture of its two enantiomers, which differ substantially in pharmacology and behavioral profile. (S)-ketamine has approximately fourfold higher affinity for the NMDA receptor than (R)-ketamine and produces more pronounced dissociative and psychotomimetic effects; it is the basis of the only FDA-approved intranasal formulation (Spravato) for treatment-resistant depression. (R)-ketamine has lower NMDA receptor affinity and, in preclinical models, has demonstrated antidepressant-like effects with a more favorable side-effect profile and potentially lower abuse liability (38).

From a substance use disorder perspective, enantiomeric divergence has two implications. First, the greater reinforcing properties of (S)-ketamine relative to (R)-ketamine in preclinical self-administration models suggest that the enantiomeric composition of the therapeutic preparation may modulate abuse liability (38). Second, the emerging clinical interest in (R)-ketamine as a potentially safer antidepressant with lower dissociative burden may extend to SUD indications, although there is a lack of clinical SUD trials of isolated (R)-ketamine.

### 3.4 Opioid receptor involvement

In addition to its glutamatergic actions, ketamine interacts with opioid receptors. Levinstein et al. demonstrated that ketamine’s reinforcing properties and its anti-craving effects involve bifunctional modulation of both NMDA and opioid (μ and κ) receptors, and that the relative contribution of each receptor system may vary by dose and context (56). Janssen-Aguilar et al., in their systematic review of 14 clinical ketamine-in-SUD studies, similarly characterized ketamine’s anti-craving action as involving both NMDA receptor antagonism and secondary opioid receptor modulation, making its mechanism comparable to that of acamprosate and ibogaine through the NMDA receptor pathway (57).

### 3.5 Addiction-specific mechanistic pathways

While the glutamatergic and synaptic plasticity mechanisms described in the previous section provide the molecular foundation for ketamine’s therapeutic actions, their relevance to addiction treatment is best understood through four distinct mechanistic pathways, each with different implications for clinical application and patient selection (summarized in **Table 2**).

**Table 2:** Four addiction-specific mechanistic pathways of ketamine.

Pathway	Key mechanism	Key evidence	Clinical implication
1. Synaptic plasticity/BDNF-TrkB-mTOR	Reversal of maladaptive synaptic strengthening in NAc, PFC, amygdala via BDNF-TrkB-mTOR-dependent synaptogenesis	(52, 53)	Rationale for repeated dosing to consolidate circuit remodelling
2. Memory reconsolidation interference	NMDA blockade during reconsolidation window weakens drug cue–reward memory traces	(58–60)	Timing of administration relative to cue retrieval is mechanistically critical
3. Ego dissolution/mystical experience	Ketamine-occasioned transformative experience promotes psychological flexibility and weakens drug-associated self-narrative	(61)	Therapeutic set and setting are mechanistically active, not merely supportive
4. Antidepressant/anti-anhedonic carryover	Rapid relief of comorbid depression/anhedonia removes negative-affect driver of relapse	(11, 39)	Stratify trials by baseline depression; comorbid patients may show larger effect

NAc, nucleus accumbens; PFC, prefrontal cortex; BDNF, brain-derived neurotrophic factor; TrkB, tropomyosin receptor kinase B; mTOR, mechanistic target of rapamycin; NMDA, N-methyl-D-aspartate.

#### 3.5.1 Synaptic plasticity and BDNF-TrkB-mTOR signaling in addiction circuits

As outlined in Section 3.1, ketamine’s NMDA receptor antagonism triggers a cascade of downstream neuroplastic changes via BDNF release, TrkB receptor activation, and mTOR-dependent protein synthesis, leading to rapid synaptogenesis. In the addiction context, the critical framing is not the reversal of stress-induced synaptic deficits in depression, but rather the disruption of maladaptive synaptic strengthening within mesocorticolimbic circuits. Repeated drug use consolidates drug-related cue–reward associations through long-term potentiation-like mechanisms in the nucleus accumbens, basolateral amygdala, and prefrontal cortex. These strengthened synaptic engrams underlie conditioned craving responses to drug-associated cues, context-triggered relapse, and the compulsive quality of drug seeking despite adverse consequences (62).

Ketamine’s rapid BDNF-TrkB-mTOR-dependent synaptogenesis may reverse these pathological engrams by promoting homeostatic synaptic remodeling and restoring prefrontal inhibitory control over subcortical reward circuits. This provides a mechanistic rationale for the observed effects of ketamine on craving reduction and abstinence that is independent of any antidepressant action (52).

#### 3.5.2 Memory reconsolidation interference

Another mechanistic pathway supported by direct clinical evidence in addiction is the disruption of memory reconsolidation. According to reconsolidation theory holds that consolidated memories become transiently labile and susceptible to change when reactivated, providing a narrow window during which the memory trace can be weakened or modified before re-stabilization (58, 59). Drug-associated memories, which are the conditioned links between drug cues and reward, are maintained by glutamatergic synaptic mechanisms. Additionally, NMDA receptor activity is essential for their reconsolidation after retrieval.

Ketamine’s NMDA receptor antagonism, when administered during the reconsolidation window immediately after retrieval of drug-related memories, can block the restabilization of maladaptive cue-reward associations, thereby diminishing activity. Das et al. showed clinical evidence in alcohol use disorder, indicating that a single ketamine infusion given after retrieving alcohol-related memories produced sustained reductions in drinking frequency and cravings for up to nine months, compared to retrieval alone or ketamine without retrieval (60). This memory reconsolidation paradigm is mechanistically distinct from ketamine’s acute neuroplastic effects and provides a rationale for the precise timing of ketamine administration relative to cue exposure in clinical protocols.

#### 3.5.3 Ego dissolution, mystical experience, and psychological flexibility

A third pathway involves sub-anesthetic ketamine’s psychological effects, like dissociation and ego-dissolution, which at appropriate doses and settings can lead to mystical or transformative experiences. Unlike the neurobiological pathways, this one works through psychological mechanisms: dissolving the habitual self-narrative and cognitive rigidity may foster openness to change and diminish the importance of drug-related identities.

Rothberg et al. provided the first formal mediation analysis demonstrating that mystical-type experiences occasioned by ketamine statistically mediated improvements in at-risk drinking outcomes in a randomized controlled trial: the magnitude of the mystical experience during the ketamine session predicted the degree of subsequent reduction in alcohol use, independent of the pharmacological effect on craving (61). This finding has direct implications for clinical protocol design: it indicates that the therapeutic setting, psychotherapeutic preparation, and integration support around ketamine administration are not just supplementary but are actively involved components of the intervention, mediating a significant role in mediating the treatment effect.

#### 3.5.4 Anti-anhedonic and antidepressant carryover effects

The fourth mechanistic pathway concerns the substantial overlap between addiction and depression at both the neurobiological and clinical levels. Depression and anxiety disorders co-occur in 30–60% of individuals with substance use disorders, and negative affect, dysphoria, anhedonia, and stress sensitivity are primary drivers of relapse in the opponent-process model of addiction (11, 63). For this population, ketamine’s rapid antidepressant action may contribute to SUD outcomes not through any addiction-specific mechanism, but through the rapid relief of the negative emotional states that motivate continued drug use.

This antidepressant carryover pathway has important implications for outcome attribution and patient selection. In clinical trials enrolling patients with SUD and significant comorbid depression, observed improvements in craving, abstinence, or relapse prevention may reflect antidepressant effects rather than direct disruption of addiction circuits. In patients with SUD and minimal depressive comorbidity, this pathway is likely to contribute less to overall treatment response. Future trials should stratify participants based on baseline depression severity to better understand these effects. Additionally, glutamatergic dysfunction is linked not only to depression and addiction but also to suicidality. Esketamine’s ability to rapidly normalize synaptic glutamate signaling offers a mechanistic basis for its use in patients with both SUD and depressive disorder who experience suicidal ideation (39).

## 4 Clinical evidence – ketamine

### 4.1 Alcohol use disorder: trials on craving and relapse

Clinical and experimental trials provide emerging evidence that ketamine-based interventions can reduce alcohol craving and relapse risk in alcohol use disorder.

In a Phase 2, double-blind, 2×2 factorial RCT of 96 adults with severe AUD, Grabski et al. assigned participants randomly to receive three weekly ketamine infusions (0.8 mg/kg) or saline, combined with either mindfulness-based relapse prevention (MBRP) or standard drug counseling as usual. The percentage of days abstinent showed a positive trend for ketamine at six months. However, the second primary outcome, confirmed relapse, showed no significant effect (pooled OR 0.70, 95% CI 0.28–1.75) (64). The ketamine-by-MBRP interaction was not statistically significant, and the trial was not designed to detect the commonly cited ‘largest effect with MBRP’; thus, it should be seen as an exploratory subgroup finding rather than definitive evidence of synergy. There was substantial: 100%, 95%, and 100% of ketamine recipients correctly identified their allocation in each of the three infusions, compared with 27%, 34%, and 23% of placebo recipients. The authors explicitly acknowledge this limitation. Additionally, 26% of participants reported prior recreational ketamine use, 49% psilocybin, and 44% LSD. The authors highlight this as a potential expectancy amplifier that may inflate the active-arm response (64, 65). A pilot inpatient trial of a single ketamine infusion (0.5 mg/kg) in high-utilization AUD inpatients showed lower 30−day readmission rates compared with linkage-as-usual, though the difference was not significant in this small sample (Ketamine n= 17 patients) (66).

A separate randomized trial of 40 adults with alcohol dependence receiving motivational enhancement therapy found that a single ketamine infusion (0.71 mg/kg) significantly increased daily abstinence, delayed time to relapse, and reduced heavy drinking days compared with midazolam, indicating both craving-related and relapse-related benefits over 21 days post-infusion (67). Secondary analyses of this trial show that reductions in at−risk drinking were mediated by mystical-type experiences, suggesting a psychological pathway linking ketamine’s acute subjective effects to improved alcohol outcomes (61).

Systematic reviews and meta-analyses further support these findings, showing that ketamine, especially when combined with psychotherapy, consistently reduces alcohol consumption and cravings in both clinical and non-treatment-seeking populations (68–71). For example, studies using memory reconsolidation paradigms have demonstrated that administering ketamine after retrieving maladaptive alcohol-related memories results in substantial reductions in drinking frequency and urges to drink, with effects lasting up to nine months post-intervention (32, 60). While some studies report mixed results on long-term relapse prevention and craving reduction, likely due to heterogeneity in dosing regimens, patient populations, and outcome measures, the overall trend suggests that adjunctive ketamine treatment is associated with improved confidence in abstaining from alcohol and longer periods of reduced consumption (72, 73).

Additional research on individuals with AUD has demonstrated that sublingual esketamine hydrochloride, when combined with mindfulness-based interventions, resulted in temporary reductions in alcohol cravings relative to placebo. However, there were no significant differences in long-term reductions in consumption between the groups (74). Importantly, across trials of ketamine (and esketamine), no serious adverse events or evidence of misuse were reported. In addition, both were well tolerated and capable of delaying relapse, reducing heavy drinking, and increasing abstinent days, particularly when integrated with structured psychotherapies. Nevertheless, further large-scale research is needed to clarify optimal dosing strategies, long-term safety profiles, and the most effective combinations of pharmacological and psychotherapeutic interventions to support sustained recovery in AUD. A summary of the trials is available in **Table 3**. In addition, the MORE-KARE Phase 3 trial, the largest planned ketamine-AUD study to date, is currently recruiting and represents the definitive test of whether the Phase 2 signal identified by Grabski et al. (64) is reproducible at scale with rigorous blinding controls and clinically meaningful follow-up durations.

**Table 3:** Key ketamine AUD trial outcomes.

Outcome	Ketamine effect vs control	Citations
Days abstinent (3–6 months)	↑ Days abstinent, esp. with relapse-prevention Percentage of days abstinent at 6 months: ketamine + MBRP 85.9% vs placebo + MBRP 65.0%, adjusted difference +20.9 percentage points. Confirmed relapse co-primary: OR 0.70 (95% CI 0.28–1.75), p = 0.45.	(64–66).
Time to relapse/heavy drinking	Delayed relapse, ↓ heavy drinking days Daily abstinence over 21 days showed a significant group × time interaction favoring ketamine (p < 0.05). Time to relapse significantly delayed in ketamine arm.	(61, 67)
Alcohol craving	Acute/transient craving reductions esketamine: no significant between-group difference in long-term consumption reduction.	(66, 74)

### 4.2 Ketamine on cocaine addiction

Randomized and controlled trials in cocaine addiction indicate that a subanesthetic ketamine can reduce cocaine use, craving, and relapse, particularly when paired with behavioral interventions. In a clinical RCT of 55 cocaine−dependent adults receiving mindfulness−based relapse prevention, a single ketamine infusion (0.5 mg/kg) led to markedly higher end−of−study abstinence (48.2% vs 10.7% with midazolam) and a 53% lower risk of relapse (first use or dropout) over 5 weeks; craving scores were 58% lower in the ketamine group, with good tolerability (75). A systematic review of substance use disorder trials included three ketamine RCTs in cocaine use disorder, concluding that ketamine plus psychotherapy may promote abstinence and reduce cocaine consumption (76).

Laboratory-based randomized crossover trials also show decreased cocaine use and increased abstinence markers. In non–treatment−seeking cocaine users, a single subanesthetic ketamine infusion reduced cocaine self−administration by 67% relative to baseline over >24 hours compared with midazolam, representing a significant reduction in cocaine use (77). Another double−blind crossover RCT in eight cocaine−dependent volunteers found that ketamine (0.41 and 0.71 mg/kg) significantly increased motivation to quit and produced large reductions in cue−induced craving at 24 hours, compared with lorazepam. During the 4−week follow−up, days of cocaine use dropped from 22/28 at baseline to 5/28, with several participants achieving multi−week abstinence (78). Across RCTs and controlled laboratory studies, ketamine consistently reduces cocaine use, increases abstinence rates, and lowers craving in the short to medium term, especially when combined with mindfulness-based relapse prevention. Evidence is promising but based on small samples. Thus, larger, longer trials are needed to confirm the potential use of Ketamine in cocaine abuse. The summary of RCTs and controlled lab studies of Ketamine in Cocaine Use Disorder is presented in **Table 4**.

**Table 4:** Outcomes from ketamine RCTs and controlled lab studies in cocaine addiction.

Study/design & population	Ketamine dose & control	Main cocaine outcomes
Inpatient RCT + mindfulness-based relapse prevention	0.5 mg/kg vs midazolam End-of-study abstinence 48.2% (ketamine) vs 10.7% (midazolam); risk difference +37.5%; relapse risk ratio 0.47 (95% CI 0.29–0.78).	48.2% vs 10.7% abstinent; ↓ relapse risk; ↓ craving (75)
Lab crossover RCT, non–treatment-seeking users	Subanesthetic vs midazolam Cocaine self-administration reduced by 67% relative to baseline (p < 0.05 vs midazolam).	67% ↓ self-administration at >24 h post-infusion (77)
Crossover RCT, non-treatment-seeking volunteers	0.41 & 0.71 mg/kg vs lorazepam Cue-induced craving at 24 h showed large reduction (Cohen’s d > 1.0); days of use at 4-week follow-up reduced from 22/28 at baseline to 5/28.	↑ motivation to quit; large ↓ cue-induced craving; ↓ use (78)

### 4.3 Opioid use disorder and withdrawal

Clinical data on ketamine for opioid use disorder (OUD) and opioid withdrawal are limited but growing and largely preliminary. A recent scoping review identified eight clinical studies: two focused on OUD treatment and six on opioid withdrawal. Of these, only two were randomized controlled trials (RCTs), with the remainder being small case series or uncontrolled studies (79). Across these, ketamine was associated with reduced opioid craving and use and attenuation of precipitated withdrawal symptoms, often as an adjunct to buprenorphine-based protocols (55, 79). ketamine psychotherapy (KPT) treatment program for heroin dependence (double−blind, non–placebo−controlled), high−dose intramuscular ketamine (2.0 mg/kg) combined with structured psychotherapy improved 1−year abstinence compared with a lower−dose condition, and a follow−up RCT found that three KPT sessions produced higher 1−year abstinence than a single session (50% vs 22.2%), although group differences in craving were not observed (32, 55).

A critical temporal limitation of the OUD evidence base is that the most methodologically rigorous RCT to date (80) was conducted using rapid opioid antagonist induction under general anesthesia, a paradigm now rarely used in clinical practice. Since 2006, buprenorphine-based low-dose induction, high-dose buprenorphine bridging, and extended-release naltrexone have become the main pharmacotherapeutic approaches for OUD, supported by substantially improved evidence of safety and retention (81, 82). The extent to which ketamine’s observed attenuation of precipitated withdrawal in that earlier protocol translates into adjunctive benefit within modern buprenorphine-centered care pathways remains unknown and untested. More recent OUD-specific data, including the scoping review by Shen et al. (79) and the pharmacotherapy review by Onisiforou et al. (55), suggest plausible mechanisms for ketamine’s utility in opioid craving and withdrawal, but are based on small, heterogeneous studies that do not align with current standard-of-care protocols. Larger trials, specifically integrated into modern buprenorphine-based frameworks, are necessary before any translational conclusions can be made.

For opioid withdrawal, an RCT of 58 opioid-dependent patients undergoing rapid antagonist-induced withdrawal under general anesthesia, ketamine (0.5 mg/kg/h) was compared with saline. The study found lower objective withdrawal scores during and immediately after antagonist induction in the ketamine group, along with reduced cardiovascular and cortisol responses and a decreased need for adjunctive medications. However, abstinence rates at 4 months did not differ between groups (80). Systematic and narrative reviews conclude that ketamine may attenuate acute withdrawal and support abstinence when paired with intensive psychotherapy, yet overall evidence in OUD is judged inconclusive due to small samples, lack of placebo controls in key trials, and limited long−term follow−up (32, 57, 76, 83, 84). Current guidance emphasizes the need for larger, well−controlled RCTs before ketamine can be recommended for attenuating OUD beyond experimental or highly specialized settings (32, 55, 76, 79). Across the few RCTs, ketamine shows signals for reducing acute withdrawal severity and enhancing longer−term abstinence when combined with intensive psychotherapy. Evidence remains preliminary, supporting ketamine as an experimental rather than established option in OUD and withdrawal management. The summary of the study outcomes of Ketamine in OUD is presented in **Table 5**.

**Table 5:** Key RCT outcomes of ketamine in OUD and withdrawal.

Study/population	Ketamine regimen & comparator	Primary OUD/withdrawal outcomes	Main findings
Heroin dependence, detoxified inpatients (KPT dose-comparison RCT)	Single high-dose IM ketamine 2.0 mg/kg + structured psychotherapy vs low-dose control session	Abstinence, craving, mood over 1 year	Higher 1-year abstinence with high-dose KPT; improvements in abstinence without clear between-group craving differences 1-year abstinence 50% (3 sessions) vs 22.2% (1 session), reported as statistically significant (55)
Heroin dependence, detoxified inpatients (1 vs 3 KPT sessions RCT)	1 vs 3 sessions of 2.0 mg/kg IM ketamine + psychotherapy	1-year abstinence, craving, anxiety, depression	Three sessions: 50% abstinent vs 22.2% with one session; no significant group differences in craving, anxiety, or depression 1-year abstinence 50% (3 sessions) vs 22.2% (1 session), reported as statistically significant (55)
Opioid-dependent adults undergoing rapid naltrexone induction under GA	Ketamine 0.5 mg/kg/h IV vs saline during anesthesia	Withdrawal severity (OOWS/SOWS), physiological stress, 4-month abstinence	Lower withdrawal scores, BP, pulse, cortisol; less adjunctive meds in ketamine group; no difference in 4-month abstinence Objective withdrawal scores significantly lower in ketamine group during induction (p < 0.05); 4-month abstinence: no significant difference between groups (80)

### 4.4 Ketamine for treatment-resistant depression: recent updates

Although TRD falls outside the primary scope of this review, evidence from TRD trials and real-world TRD cohorts: (1) FDA (2019) and EMA (2019) (85, 86) approval of ketamine for TRD via esketamine (Spravato) provides the only supervised, Risk Evaluation and Mitigation Strategy (REMS)-governed delivery model against which SUD protocols; (2) long-term TRD safety data provide the most systematically collected pharmacovigilance data on repeated ketamine exposure (87); and (3) many SUD patients also have depression, making TRD evidence highly relevant for clinical decision-making (88).

Recent evidence confirms ketamine (IV, oral, subcutaneous, intranasal) and intranasal esketamine as rapidly acting treatments for treatment−resistant depression (TRD), with growing data on long−term and maintenance use. Regarding the efficacy and durability of the response, a meta−analysis of dose−escalation that included 12 randomized controlled trials (RCTs) found large antidepressant effects for IV ketamine (Hedges g = 1.52) and smaller but significant effects for intranasal esketamine (g = 0.31). IV efficacy plateaued above 0.5 mg/kg, and the optimal esketamine dose was 56–84 mg (89). Another systematic review included 28 RCTs showed strong effects within 4 h of ketamine infusion, peaking at 24 h, and still present at 7 days; multiple infusions enhanced and prolonged the ketamine benefit (90). In addition, a randomized, double-blind, active-controlled trial revealed that subcutaneous flexible-dose ketamine administered twice weekly for 4 weeks achieved significantly higher remission rates in TRD than midazolam (19.6% vs 2.0%) (91). Another phase 2 clinical trial found that extended-release oral ketamine tablets administered twice weekly for 12 weeks showed a significant effect compared with placebo on the Montgomery-Asberg Depression Rating Scale (MADRS) at week 13, with dose-dependent relapse reduction (placebo 70.6% vs 42.9% at 180 mg) (92).

Blinding integrity is essential for valid placebo-controlled studies, yet it is systematically compromised in ketamine trials due to the drug’s clear dissociative and perceptual effects. A critical and frequently underappreciated methodological limitation across ketamine TRD trials is a major reason for blinding failure. Active-arm participants reliably identify their allocation because of ketamine’s distinctive dissociative effects, yielding expectancy-inflated, placebo-subtracted effect sizes. Two recent studies provide the most rigorous quantification of this problem to date. Lii et al., using a surgical anaesthesia masking paradigm, demonstrated that the accuracy with which patients guessed their allocation, irrespective of actual treatment received, was itself a significant predictor of MADRS scores at day 14, providing direct evidence that expectancy, rather than pharmacological action alone, drives a meaningful proportion of the observed antidepressant signal (93). The KARMA-Dep 2 RCT reported a null result for its primary MADRS outcome, alongside documented unblinding of both patients and raters, offering the clearest demonstration yet that, when expectancy is accounted for, the placebo-corrected effect size may approach zero (94). In the KADS subcutaneous ketamine RCT, functional unblinding rates were not formally assessed. The Grabski et al. (64) AUD trial documented near-complete functional unblinding, as detailed in Section 4.1. Taken together, these findings reinforce the recommendation that future trials include active comparators with matched subjective profiles, such as sub-dissociative midazolam or low-dose ketamine, and formally measure unblinding rates as a co-primary methodological outcome, to yield interpretable placebo-controlled effect estimates applicable to both TRD and SUD indications.

### 4.5 Ketamine risk profile: dissociation, psychosis, and dependency

Ketamine’s growing use for depression and substance use disorders raises concerns about dissociation, psychosis in vulnerable patients, and dependence, especially with repeated dosing in people with histories of drug abuse. Across clinical trials, acute dissociative and psychotomimetic effects (e.g., depersonalization, hallucinations) are common but typically transient, peaking within 30–60 minutes of infusion and resolving within 1–2 hours (42, 87, 88, 95). In treatment−resistant depression, dissociation and psychotic−like symptoms were usually mild to moderate and rarely led to discontinuation; however, an observational study showed greater and more fluctuating psychotic scores in patients with epilepsy (95). The principal psychiatric contraindication to ketamine is a personal or first-degree family history of a primary psychotic disorder, due to ketamine’s pro-psychotomimetic acute effects and the theoretical risk of precipitating or accelerating the onset of psychosis in genetically predisposed individuals (96, 97).

Regarding addiction and dependence, ketamine’s dissociative effects and overlap with reward circuitry clearly confer abuse liability, demonstrated by extensive preclinical self−administration data and human recreational use (38, 43, 98, 99). Scoping and narrative reviews emphasize that long−term clinical data are limited and that repeated dosing could theoretically promote sensitization and substance use disorder, particularly among patients already treated for addiction (43, 88, 96, 100). Surveys of real-world sublingual and intranasal ketamine used in the treatment of depression indicate low average levels of drug craving. However, they recommend cautious prescribing, continuous monitoring for dose escalation, and screening for prior substance use disorders (SUDs) (43, 101). Overall, ketamine may be administered safely within structured protocols; nonetheless, in patients with histories of substance abuse, clinicians must carefully weigh the benefits against the significant risks of dissociation, psychosis in susceptible individuals, and the potential for dependency with repeated exposure.

## 5 Abuse risks and safety concerns

### 5.1 Ketamine adverse events and toxicity

Ketamine’s adverse event and toxicity profile is complex, with risks that vary by dose, frequency, and patient vulnerability. Acute adverse effects most commonly include dissociation, perceptual disturbances (such as hallucinations or derealization), and transient cardiovascular changes, such as elevated blood pressure and tachycardia. These effects typically peak within an hour of administration and resolve within a few hours, but they can be distressing or dangerous in certain populations—especially those with underlying psychiatric or cardiovascular conditions (102–106). Psychiatric symptoms, including agitation, anxiety, and psychotomimetic reactions, are well documented, particularly at higher doses or in recreational settings (97, 102). Cardiovascular stimulation is generally mild but can be significant in susceptible individuals; rare cases of laryngospasm and respiratory depression have been reported, especially when ketamine is combined with other CNS depressants (103, 107). With repeated therapeutic exposure, patient distress and perceived severity of dissociative effects may diminish through habituation and optimized pre-medication and integration support; however, the magnitude of blood pressure elevation, heart rate increase, and dissociation does not reliably attenuate, which is precisely why the Spravato (esketamine) REMS mandates a minimum two-hour supervised observation period with vital-sign monitoring after every dose regardless of treatment duration (87, 102).

Chronic toxicity is primarily characterized by ketamine-induced uropathy (bladder dysfunction), which manifests as cystitis, urinary frequency/urgency, hematuria, and, in severe cases, hydronephrosis or renal impairment, especially among frequent recreational users or those on high-dose/prolonged period of prescription (108–110). Neurological effects include persistent cognitive deficits (notably in working and episodic memory), executive dysfunction, and mood disturbances after long-term or high-frequency use. These effects are less common at therapeutic doses but have been observed in chronic pain and psychiatric populations receiving repeated infusions (51, 87, 111, 112).

Hepatotoxicity has emerged as a concern with repeated or continuous administration: case series reports elevations in liver enzymes, cholangitis, biliary dilation, and even hepatic failure, more prevalent with higher cumulative exposure (50, 113–115). Other organ system effects include gastrointestinal symptoms (abdominal pain), potential for psychological dependence (especially at high doses), and rare reports of sclerosing cholangitis or upper GI pathology (116, 117). Overall, the risk of serious adverse events is low when ketamine is used intermittently at subanesthetic doses for medical indications; however, dose-dependent toxicity profiles underscore the need for careful patient selection, monitoring of urinary/liver function during prolonged therapy, and vigilance for neuropsychiatric complications, particularly in vulnerable groups or those with histories of substance abuse (51, 111, 118, 119). The summary of the Ketamine acute and chronic toxicity domains is presented in **Table 6**.

**Table 6:** Ketamine acute and chronic toxicity domains.

Domain/theme	Key findings
Acute CNS & perceptual effects	Dissociation, feeling “strange/weird/loopy,” hallucinations, anxiety, dizziness; usually resolve ≤2 h (102–106).
Acute cardiovascular effects	Transient ↑ blood pressure and heart rate; cardiovascular tone generally stimulated, serious events rare (103, 107).
General acute toxicity profile	Wide range of neuro-behavioral effects (agitation, confusion), vomiting, emergence phenomena (97, 102).
Chronic uropathy	Ketamine-induced ulcerative cystitis, severe storage symptoms, pelvic pain, contracted bladder,hydronephrosis; can extend to ureter/kidney (108–110).
Neurological/cognitive impacts	Long-term use linked to neurocognitive impairment (memory, executive deficits), schizophrenia-like syndromes (51, 87, 111, 112, 116, 117).
Gastrointestinal & hepatobiliary	Abdominal pain, abnormal liver function tests, cholangiopathy/cholangiopathies with prolonged/chronic exposure (50, 113–115).
Dose– and exposure–dependence	Most adverse effects are dose-related; chronic high-dose/recreational use drives uropathy,cognitive and hepatobiliary toxicity, while short-term therapeutic trials show few persistent organ toxicities (51, 111, 118, 119).

### 5.2 Ketamine’s abuse liability

Recreational patterns of ketamine use, particularly among younger age groups, are typically characterized by repeated high-dose administration, often in combination with other substances, driven by the drug’s dissociative and perceptual effects. This pattern of use carries a well-documented risk of psychological dependence, tolerance, ulcerative cystitis, and neurocognitive impairment that is substantially greater than in supervised clinical programs (106, 108, 120, 121).

In contrast, scoping and systematic reviews of ketamine administered for treatment−resistant depression under supervised, time−limited protocols report that therapeutic subanesthetic doses rarely lead to misuse, dependence, or diversion, with only a very small minority of patients showing clear tolerance or dependence across thousands of treated individuals (41, 43, 51).

Comparative pharmacokinetic analyses indicate that cumulative ketamine exposure among heavy recreational users exceeds that in clinical programs by more than 90-fold compared with the cumulative dose typical of a 6-month esketamine TRD maintenance schedule under REMS supervision. This likely explains why dependence, ulcerative cystitis, and severe neuropsychiatric complications are concentrated in the recreational population and have not emerged as major signals in structured therapeutic use to date (41, 51, 109). Nonetheless, the rapidly expanding availability of ketamine outside specialist centers, including less−supervised non−parenteral formulations, raises concern about diversion from medical settings, self−management of dosing, and access among people with existing substance use disorders (44, 122–124).

On a cumulative milligram basis, a heavy recreational ketamine user consuming 1–2 g per day accumulates approximately 30–60 g per month (108, 120, 125), compared with a total course dose of 35–168 mg in a typical therapeutic substance use disorder trial protocol using intravenous infusions, or approximately 1,680–3,360 mg of esketamine in a 6-month treatment-resistant depression maintenance schedule under REMS supervision (64, 75, 87). This represents a 90 to 200-fold difference in cumulative exposure when comparing heavy recreational use with the esketamine TRD maintenance comparator, and a substantially larger difference when comparing with a single SUD trial course (51, 109).

This dose differential provides the most plausible pharmacological explanation for the striking divergence in adverse event profiles between therapeutic and recreational ketamine use. Ketamine-induced ulcerative cystitis, hepatobiliary toxicity, and severe neurocognitive impairment are concentration- and duration-dependent toxic effects consistently associated with heavy recreational use patterns but have not emerged as major safety signals in structured clinical programs, where cumulative exposure remains orders of magnitude lower (51, 109). It is therefore the total cumulative dose, route of administration, and absence of clinical supervision, rather than the molecule itself, that drive the harm differential between therapeutic and recreational use. A dose Comparison summary between therapeutic versus recreational ketamine Exposure is available in **Table 7**.

**Table 7:** Illustrative dose comparison: therapeutic versus recreational ketamine exposure.

Setting/population	Route of administration	Dose per session	Frequency	Estimated cumulative dose	Reference(s)
Therapeutic use — supervised clinical settings					
Esketamine (Spravato) — TRD acute phase (REMS)	Intranasal	56–84 mg per session	Twice weekly for 4 weeks (8 sessions)	∼448–672 mg over 4 weeks	(126)
Esketamine (Spravato) — TRD maintenance (REMS)	Intranasal	56–84 mg per session	Every 1–2 weeks (maintenance phase)	∼1,680–3,360 mg over 6 months (∼840–1,680 mg racemic ketamine equivalent)	(87, 126)
IV racemic ketamine — SUD trials (e.g. AUD, cocaine use disorder)	Intravenous	0.5–0.8 mg/kg per infusion (∼35–56 mg for a 70 kg patient)	1–3 infusions over 1–3 weeks	∼35–168 mg total across a full trial course	(64, 75)
IV racemic ketamine — TRD (off-label infusion clinics)	Intravenous	0.5 mg/kg per infusion (∼35 mg for a 70 kg patient)	Six infusions over 2–3 weeks (standard course)	∼210 mg over 2–3 weeks	(42, 88)
Subcutaneous ketamine — TRD (KADS trial)	Subcutaneous	Flexible dose up to 0.5 mg/kg (∼35 mg for a 70 kg patient)	Twice weekly for 4 weeks (8 sessions)	∼280 mg over 4 weeks	(91)
Recreational/non-medical use					
Recreational use — occasional/social users	Intranasal (most common); oral; intramuscular	100–500 mg per session (user-reported)	Weekly or less	∼400–2,000 mg per month	(108, 127)
Recreational use — heavy/frequent users	Intranasal; intramuscular; oral	500 mg–2,000 mg per session (user-reported)	Daily to multiple times daily	∼15,000–60,000 mg per month (15–60 g/month)	(51, 108)
Ketamine use disorder — severe cases	Intranasal; intramuscular	Up to several grams per day (“use until supplies run out” pattern)	Continuous/compulsive	>60,000 mg per month in severe cases	(120, 125)
Cumulative dose differential summary					
Fold difference: heavy recreational vs esketamine TRD maintenance (6 months)	—	—	—	Heavy recreational (∼180,000–360,000 mg/6 months) vs esketamine TRD maintenance (∼1,680–3,360 mg/6 months) = approximately 90- to 200-fold differential	(51, 109)
Fold difference: heavy recreational vs IV ketamine SUD trial course	—	—	—	Heavy recreational (∼15,000–60,000 mg/month) vs SUD trial course (∼35–168 mg total) = approximately 90- to 1,700-fold differential depending on comparator	(64, 108)

TRD, treatment-resistant depression; SUD, substance use disorder; AUD, alcohol use disorder; IV, intravenous; REMS, Risk Evaluation and Mitigation Strategy.

Emerging surveillance data highlight a worsening trajectory of recreational harm. In England, Wales, and Northern Ireland, ketamine-related deaths rose substantially between 1999 and 2024, with the most recent years showing accelerating mortality, as documented by Pullen et al. in an update report (128). Concurrently, the number of individuals entering treatment for ketamine use disorder in the UK increased more than 10-fold over the past decade, reflecting both rising prevalence and growing clinical recognition of the condition (120, 129). These epidemiological trends are directly relevant to the abuse liability framing of this review and underscore that the recreational harm signal is not static.

Recent reviews and recommendations therefore stress that while the intrinsic abuse liability of ketamine is moderate and comparable to other controlled psychotropics, real−world misuse risk is shaped by factors such as prior SUD history, ease of access to ketamine from non-prescription sources, and weak monitoring systems, requiring careful screening, supervision, and regulation in therapeutic use (44, 97, 116, 122–124). A summary for contrasting dependence patterns in medical and recreational ketamine use is provided in **Table 8**.

**Table 8:** Contrasting dependence patterns in medical and recreational ketamine use.

Aspect	Therapeutic use (TRD, supervised)	Recreational / KUD	Citations
Liking/craving	Often neutral/mixed; cravings rare	Strong liking for dissociation; frequent craving	(41, 101, 123, 125, 129–131)
Dose pattern	Fixed or modestly titrated; no typical escalation	Marked escalation (up to multi-gram daily).	(41, 120, 130, 131).
Dependence prevalence	Very rare cases; no consistent signal across 16 depression studies (2,174 patients); 4 patients showed clear tolerance or dependence.	8.6% of past-year users met probable dependence criteria on the (SDS threshold ≥3) in a large global survey; many report compulsive use patterns. Note: SDS-defined probable dependence is not equivalent to a formal DSM-5-TR or ICD-11 Ketamine Use Disorder diagnosis.	(41, 43, 51, 120, 127, 129)
Withdrawal pattern	None or mild, overlaps with depressive relapse	Prominent psychological abstinence symptoms	(41, 123, 129)

SDS, Severity of Dependence Scale.

### 5.3 Psychological dependence on ketamine: a comparative view of therapeutic vs. recreational use

Psychological dependence on ketamine differs markedly between supervised medical treatment and non−medical, recreational use. Key contrasts include drug liking/craving, dose escalation, compulsive patterns, and withdrawal−like experiences.

#### 5.3.1 Patterns in therapeutic/medical use.

##### 5.3.1.1 Low rates of dependence−like phenomena

Across 16 depression studies (2,174 patients), only 4 patients showed clear tolerance or dependence; most had no loss of effect or compulsive use (41). Systematic and narrative reviews conclude that, in professionally supervised TRD treatment, single or repeated ketamine/esketamine doses rarely lead to misuse, dependence, diversion, or “gateway” behavior (43, 51, 123, 130).

##### 5.3.1.2 Drug liking and craving

In community TRD patients receiving sublingual or intranasal ketamine, drug liking and craving were highly variable; many were neutral or negative, and dose increases mainly reflected clinical titration rather than abuse (131). In addition, an acute esketamine course showed mostly neutral liking and no cravings, with no systematic increase over 8 sessions (130).

##### 5.3.1.3 Withdrawal−like symptoms

Two clinical studies of depression found no specific ketamine withdrawal syndrome; transient dysphoria or anxiety after discontinuation was indistinguishable from the return of depressive symptoms (41).

#### 5.3.2 Patterns in recreational/non−medical use

##### 5.3.2.1 Strong psychological dependence and compulsive use

Heavy recreational users frequently report cravings, high tolerance, and use “until supplies run out,” resembling stimulant-type dependence without clear physical withdrawal (125, 132). In a large global survey of past-year ketamine users (n = 4,151), 8.6% met probable dependence criteria using the Severity of Dependence Scale (SDS threshold ≥3) (127).

##### 5.3.2.2 Motivational profile

Recreational users are driven by dissociation, psychedelic experiences, and relaxation; these effects are cited as the primary reasons for continued use and dependence (101, 120, 131).

##### 5.3.2.3 Withdrawal/abstinence symptoms

Among people with ketamine use disorder, common abstinence symptoms include craving, low mood, anxiety, irritability, and other psychological complaints (129).

## 6 The paradox: treating addiction with an addictive substance

The apparent paradox of using ketamine, a controlled drug with known potential for misuse, to treat addiction can be partly resolved by distinguishing context, dose, and intent. Clinically, ketamine is administered intermittently at subanesthetic doses within structured treatment programs, often combined with evidence−based psychotherapies such as relapse−prevention or motivational enhancement therapy, to enhance abstinence and consolidate behavior change in individuals with alcohol and other substance use disorders (61, 64, 122, 133). In this setting, systematic reviews of depression trials and real−world TRD cohorts report very low rates of clear tolerance, dependence, or craving, suggesting that, under medical supervision, ketamine’s abuse liability is comparable to that of other controlled psychotropics and far lower than in recreational use (41, 88, 122).

Neurobiologically, the same glutamatergic and opioid−modulating actions that drive reinforcement and recreational appeal also induce rapid neuroplastic changes and mystical−type experiences that can disrupt maladaptive reward memories and support abstinence, blurring the line between “side effect” and mechanism of action (36, 134) (56, 61, 133). This tension parallels the long−standing use of methadone and buprenorphine, opioid agonists with diversion and misuse risks, as first−line treatments for opioid use disorder, where structured dosing, supervision, and monitoring shift the balance decisively toward benefit (81, 82, 135, 136). Ethical analyses therefore argue that ketamine should not be rejected on the basis of its reputation but should be deployed within robust risk–benefit frameworks, emphasizing careful patient selection (e.g., caution in those with active polysubstance misuse or seeking psychoactive effects), shared decision−making, clear functional goals, and ongoing monitoring for misuse and diversion (41, 101, 122, 137, 138). In this model, what distinguishes therapeutic use from abuse is not the molecule itself, but the clinical frame: indication, dosing schedule, integration with psychotherapy, and safeguards proportionate to its known risks.

The parallel with methadone and buprenorphine is instructive as a regulatory analogy demonstrating that controlled substances with established abuse potential can be safely deployed therapeutically within appropriate governance frameworks, but it is misleading as a mechanistic analogy. Methadone and buprenorphine act at the same mu-opioid receptor as the drugs of addiction they treat, making them substitution therapies in the strict pharmacological sense. Ketamine, by contrast, is being used to treat addiction to substances primarily alcohol and cocaine that do not share its principal receptor target, the NMDA receptor. Ketamine’s therapeutic action in substance use disorders is therefore circuit-level and neuroplastic rather than substitutive, which both distinguishes it from opioid agonist therapy and limits the predictive value of that analogy for understanding ketamine’s abuse liability profile in the addiction treatment context (56, 136).

The contemporary regulatory environment for ketamine has been shaped by a series of significant events that any clinical or policy discussion must acknowledge. In October 2023, the FDA issued a safety alert regarding the risks of compounded oral and non-FDA-approved ketamine products, warning that the proliferation of telehealth-based and at-home ketamine services had created settings without the REMS-equivalent supervision that governs esketamine (Spravato) administration (124). That same month, the death of a well-known actor from acute ketamine toxicity placed ketamine’s misuse risk at the center of public and regulatory discourse in the United States.

DEA telehealth controlled-substance prescribing flexibilities, originally extended during the COVID-19 public health emergency, have been continued through 31 December 2026; simultaneously, DEA proposed special registration rules issued on 15 January 2025 specifically identified ketamine, tramadol, and benzodiazepines as high-risk substances warranting additional safeguards. Despite these domestic pressures, ketamine retains its DEA Schedule III classification (unchanged since 1999) and remains unscheduled under the 1971 UN Convention on Psychotropic Substances, an outcome that reflects the WHO Expert Committee on Drug Dependence’s repeated recommendation against international scheduling, most recently on grounds that the public health benefits of medical use outweigh the harms of recreational use when appropriate controls are in place (124, 137).

The gap between the controlled conditions of clinical trials and the heterogeneous reality of off-label ketamine delivery outside specialist centers is substantial and constitutes a major patient safety concern. Real-world ketamine clinics vary widely in staffing, with many operating without trained psychotherapists, without the REMS-equivalent vital-sign monitoring required for esketamine, without structured long-term follow-up, and without systematic screening for emerging uropathic or hepatobiliary signals. The rapid scale-up of telehealth and at-home ketamine services has amplified this risk by enabling access among individuals with active polysubstance use disorders or contraindicated psychiatric histories who may not be identified without face-to-face pre-screening. Until regulatory standards for off-label clinical practice are established and enforced, the risk–benefit calculus favoring ketamine in well-controlled trials cannot be assumed to apply to real-world delivery (122, 124).

## 7 Conclusion

**Figure 2:**
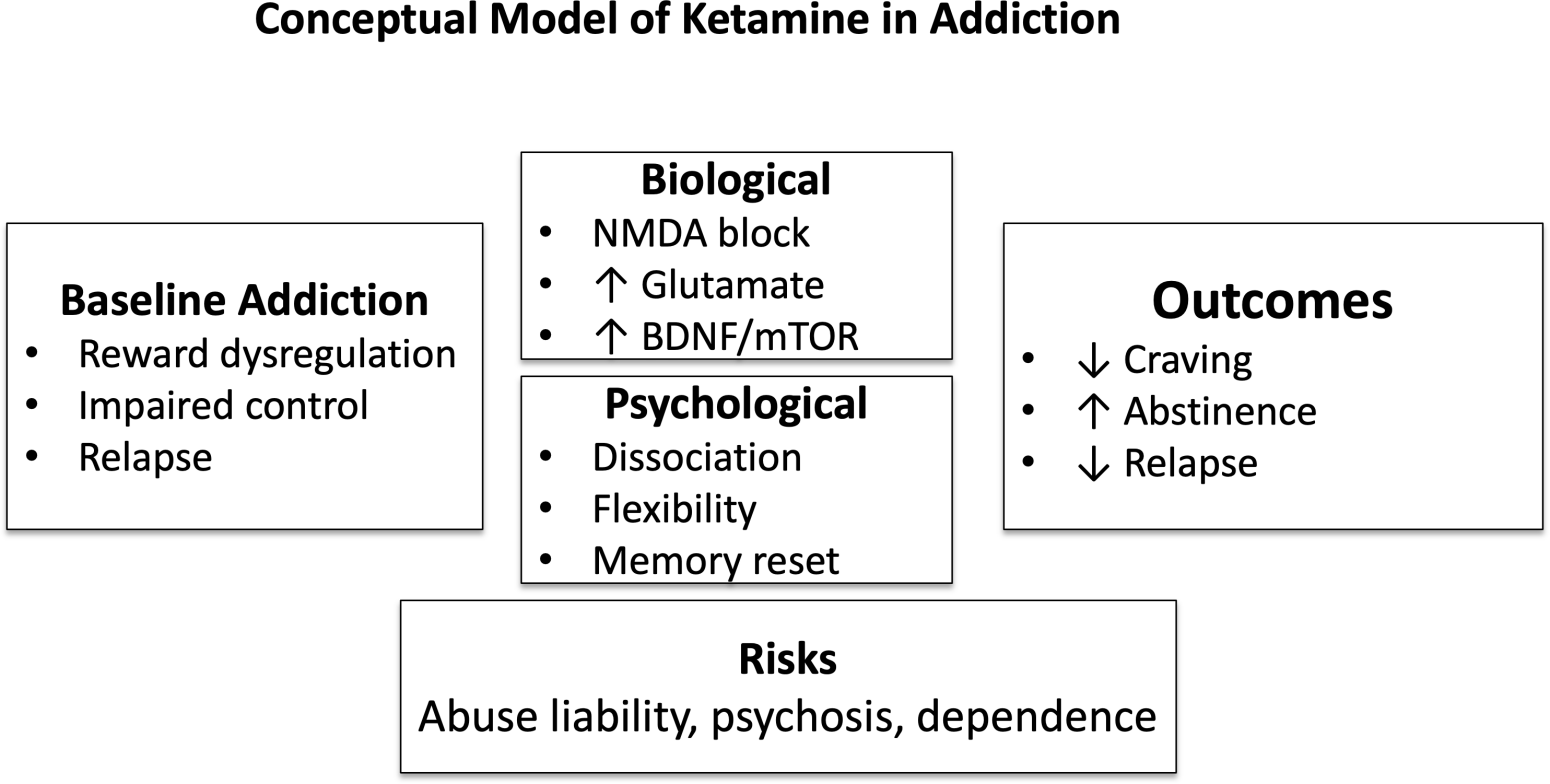
Conceptual model illustrating ketamine’s dual biological and psychological mechanisms in addiction treatment and their associated clinical outcomes and risks.

Ketamine in addiction treatment currently occupies a narrow but promising space between innovation and legitimate concerns about abuse. Across alcohol, opioid, and cocaine use disorders, early trials and systematic reviews suggest that carefully dosed, intermittently administered ketamine in supervised settings, often combined with psychotherapy, can reduce craving and increase abstinence, with some sustained benefits. Yet its dissociative and reinforcing properties confer a clear risk of abuse. Notably, existing SUD trials have not reported illicit transition, diversion, or ketamine use disorder when delivered under rigorous clinical protocols, highlighting the importance of context, dose, and monitoring, similar to other addictive pharmacotherapies such as opioid agonists. For cannabis and tobacco use disorders, there is a lack of controlled clinical data for ketamine currently; the former remains an important unaddressed indication given the absence of any approved pharmacotherapy. Overall, the evidence supports a cautiously optimistic view: ketamine may serve as an adjunct for refractory SUDs and for TRD but should remain restricted to well-controlled programs with conservative patient selection, shared decision-making, long−term safety surveillance, and confirmation in larger, methodologically robust trials before wider use. The conceptual model of ketamine in addiction is illustrated in **Figure 2**.

## 8 Future directions

Future research should prioritize large, well-powered randomized controlled trials with standardized dosing regimens, longer follow-up periods, and consistent outcome measures to clarify ketamine’s effects across different substance use disorders. Importantly, the MORE-KARE Phase 3 trial, the largest planned ketamine-AUD study to date, is currently recruiting and represents the definitive test of whether the Phase 2 signal identified by Grabski et al. (64) is reproducible at scale with rigorous blinding controls and clinically meaningful follow-up durations. In parallel, mechanistic studies should further demonstrate how ketamine-induced neuroplasticity and memory reconsolidation produce sustained behavioral change. The integration of ketamine with structured psychotherapeutic interventions also warrants systematic evaluation to determine optimal timing and frequency. Importantly, long-term safety data remain limited, highlighting the need for prospective monitoring of cognitive, urological, hepatobiliary, and dependence-related outcomes, particularly in populations with prior substance use disorders. Finally, the development of SUD-specific prescribing guidelines and regulatory frameworks for ketamine should be treated as an immediate translational priority rather than deferred until completion of Phase 3 trials, given the scale of current off-label use and the heterogeneity of real-world delivery settings.
