# Supplementary material for: Treating addiction with an addictive drug: the ketamine paradox revisited
Source: Front Psychiatry. 2026 Jul 9;17:1866092. doi: 10.3389/fpsyt.2026.1866092 (PMC13391515; doi:10.3389/fpsyt.2026.1866092)
Supplement: Supplementary file 1 [file DataSheet1.pdf]

Supplementary Table S1

SANRA Checklist and Database Search Queries

Part A: SANRA Checklist

SANRA Item	Criterion	Score (0–2)	How This Review Meets the Criterion
1	Justification for the review/relevance	**2**	The Introduction explicitly frames the unmet clinical need driving interest in ketamine for SUDs, identifies the gap between efficacy and safety syntheses in existing literature, and articulates the specific contribution of this review.
2	Literature search	**2**	Four major databases searched (PubMed/MEDLINE, Scopus, PsycINFO, Web of Science). Search date: 15 March 2026. Full Boolean queries with field tags provided in Part B of this table. Predefined inclusion/exclusion criteria applied.
3	Statement of aims / research question	**2**	Aims are explicitly stated in the Introduction (‘Current problem and research aim’ sub-section): to integrate neurobiological, clinical, and safety evidence within a unified risk–benefit framework for ketamine in SUD treatment.
4	Appropriate referencing	**2**	References are drawn from peer-reviewed primary trials, systematic reviews, and meta-analyses. All citations are listed in the manuscript reference list. No unpublished data or grey literature are cited without explicit labelling.
5	Scientific reasoning	**2**	Evidence is synthesised thematically across neurobiological mechanism, clinical efficacy by substance class, and safety/abuse liability domains. Limitations of individual studies (sample size, blinding failure, temporal relevance) are explicitly acknowledged throughout.

SANRA Item	Criterion	Score (0–2)	How This Review Meets the Criterion
6	Presentation of data	**2**	Findings are organised into clearly labelled sections with supporting tables (Tables 1–8). Effect sizes, confidence intervals, and p-values are reported for primary trials where available. Directional summaries are clearly labelled as such where quantitative data are unavailable.
**Total**		**12 / 12**	**All six SANRA criteria met**

The Scale for the Assessment of Narrative Review Articles (SANRA)

Part B: Database-Specific Boolean Search Queries
Search conducted: 15 March 2026. Date range: 1 January 2010 – 15 March 2026. Language restriction: English only. Queries are presented as submitted to each database. Field tags are database-specific.

Block	Search terms
**B1. PubMed (MEDLINE) — search executed 15 March 2026**	
**Intervention**	(ketamine[MeSH Terms] OR ketamine[tiab] OR esketamine[tiab] OR "S-ketamine"[tiab] OR "R-ketamine"[tiab] OR arketamine[tiab] OR hydroxynorketamine[tiab] OR "HNK"[tiab] OR "(2R,6R)-HNK"[tiab] OR "ketamine-assisted psychotherapy"[tiab] OR "KAP"[tiab] OR "ketamine psychotherapy"[tiab] OR "KPT"[tiab])
**Condition**	("substance use disorder"[MeSH Terms] OR "substance use disorder"[tiab] OR "alcohol use disorder"[tiab] OR "alcohol dependence"[tiab] OR "cocaine use disorder"[tiab] OR "cocaine dependence"[tiab] OR "opioid use disorder"[tiab] OR "opioid dependence"[tiab] OR "heroin addiction"[tiab] OR "stimulant use disorder"[tiab] OR "cannabis use disorder"[tiab] OR "drug addiction"[tiab] OR craving[tiab] OR relapse[tiab] OR abstinence[tiab])
**Context**	(treatment[tiab] OR therapy[tiab] OR intervention[tiab] OR "clinical trial"[tiab] OR efficacy[tiab] OR effectiveness[tiab] OR therapeutic[tiab] OR "abuse potential"[tiab] OR "abuse liability"[tiab] OR "mechanism"[tiab] OR neurobiology[tiab] OR pharmacology[tiab] OR neuroplasticity[tiab] OR "memory reconsolidation"[tiab] OR "NMDA antagonist"[tiab] OR glutamatergic[tiab] OR AMPA[tiab] OR BDNF[tiab] OR "self-administration"[tiab] OR uropathy[tiab] OR cystitis[tiab] OR bladder[tiab] OR hepatotoxicity[tiab] OR dependence[tiab] OR dissociation[tiab] OR cholangiopathy[tiab])

Block	Search terms
**Date filter**	"2010/01/01"[PDAT] : "2026/03/15"[PDAT]
**Combined query**	Block 1 (intervention) and block 2 (condition) and block 3 (context) and date filter
**B2. Scopus — search executed 15 March 2026**	
**Intervention**	TITLE-ABS-KEY (ketamine OR esketamine OR "S-ketamine" OR "R-ketamine" OR arketamine OR hydroxynorketamine OR HNK OR "(2R,6R)-HNK" OR "ketamine-assisted psychotherapy" OR "ketamine psychotherapy" OR KAP OR KPT)
**Condition**	TITLE-ABS-KEY ("substance use disorder" OR "alcohol use disorder" OR "alcohol dependence" OR "cocaine use disorder" OR "opioid use disorder" OR "heroin addiction" OR "stimulant use disorder" OR "cannabis use disorder" OR craving OR relapse OR abstinence OR "drug addiction")
**Context**	TITLE-ABS-KEY (treatment OR therapy OR intervention OR "clinical trial" OR efficacy OR effectiveness OR therapeutic OR "abuse potential" OR "abuse liability" OR neuroplasticity OR "memory reconsolidation" OR "NMDA antagonist" OR glutamatergic OR AMPA OR BDNF OR "self-administration" OR uropathy OR cystitis OR bladder OR hepatotoxicity OR dependence OR dissociation OR cholangiopathy)
**Date filter**	PUBYEAR > 2009 AND PUBYEAR < 2027
**Combined query**	Block 1 and block 2 and block 3 and date filter
**B3. PsycINFO (via APA PsycNet / Ovid) — search executed 15 March 2026**	
**Intervention**	(ketamine OR esketamine OR "S-ketamine" OR "R-ketamine" OR arketamine OR hydroxynorketamine OR HNK OR "(2R,6R)-HNK" OR "ketamine-assisted psychotherapy" OR "ketamine psychotherapy" OR KAP OR KPT). ti,ab,kw.
**Condition**	("substance use disorder" OR "alcohol use disorder" OR "alcohol dependence" OR "cocaine use disorder" OR "opioid use disorder" OR "heroin addiction" OR "stimulant use disorder" OR "cannabis use disorder" OR craving OR relapse OR abstinence OR "drug addiction"). ti,ab,kw.
**Context**	(treatment OR therapy OR intervention OR "clinical trial" OR efficacy OR effectiveness OR therapeutic OR "abuse potential" OR "abuse liability" OR neuroplasticity OR "memory reconsolidation" OR "NMDA antagonist" OR glutamatergic OR AMPA OR BDNF OR "self-administration" OR uropathy OR cystitis OR bladder OR hepatotoxicity OR dependence OR dissociation OR cholangiopathy).ti,ab,kw.
**Date filter**	Publication year: 2010–2026

Block	Search terms
**Combined query**	Block 1 AND Block 2 AND Block 3 AND Date filter
**B4. Web of Science Core Collection — search executed 15 March 2026**	
**Intervention**	TS = (ketamine OR esketamine OR "S-ketamine" OR "R-ketamine" OR arketamine OR hydroxynorketamine OR HNK OR "(2R,6R)-HNK" OR "ketamine-assisted psychotherapy" OR "ketamine psychotherapy" OR KAP OR KPT)
**Condition**	TS = ("substance use disorder" OR "alcohol use disorder" OR "alcohol dependence" OR "cocaine use disorder" OR "opioid use disorder" OR "heroin addiction" OR "stimulant use disorder" OR "cannabis use disorder" OR craving OR relapse OR abstinence OR "drug addiction")
**Context**	TS = (treatment OR therapy OR intervention OR "clinical trial" OR efficacy OR effectiveness OR therapeutic OR "abuse potential" OR "abuse liability" OR neuroplasticity OR "memory reconsolidation" OR "NMDA antagonist" OR glutamatergic OR AMPA OR BDNF OR "self-administration" OR uropathy OR cystitis OR bladder OR hepatotoxicity OR dependence OR dissociation OR cholangiopathy)
**Date filter**	Publication Years: 2010–2026
**Combined query**	Block 1 AND Block 2 AND Block 3 AND Date filter
